# Glycemic index and glycemic load of brief sugary sweets: randomized controlled trials of eight Thai desserts

**DOI:** 10.3389/fnut.2024.1452602

**Published:** 2024-10-30

**Authors:** Nuttaphat Namjud, Sayamon Senaprom, Thunnicha Ondee, Akkarach Bumrungpert, Julia Heath, Krit Pongpirul

**Affiliations:** ^1^Center of Excellence in Preventive and Integrative Medicine and Department of Preventive and Social Medicine, Faculty of Medicine, Chulalongkorn University, Bangkok, Thailand; ^2^Dhurakij Pundit University, Bangkok, Thailand; ^3^Department of International Health, Johns Hopkins Bloomberg School of Public Health, Baltimore, MD, United States; ^4^Bumrungrad International Hospital, Bangkok, Thailand; ^5^Department of Infection Biology & Microbiomes, Faculty of Health and Life Sciences, University of Liverpool, Liverpool, United Kingdom

**Keywords:** glycemic index, glycemic load, Thai desserts, sugar consumption, nutritional analysis, carbohydrate content, food safety

## Abstract

**Background:**

Thai desserts, celebrated for their exquisite sweetness, are widely enjoyed for personal indulgence and as cherished souvenirs. However, their high sugar content raises concerns regarding health impacts. This study aimed to quantify the glycemic index (GI) and glycemic load (GL) in healthy volunteers following consumption of various Thai desserts, out of 10 renowned desserts from across Thailand, identified by the Tourism Authority of Thailand, characterized by differing sugar levels.

**Method:**

Eight were selected based on the absence of preservatives and microbial or chemical contaminations. Each participant consumed a 50-g serving of available carbohydrate (50avCHO) from these desserts. Ninety-six healthy volunteers, with a mean age of 31.8 ± 5.7 years, a mean body weight of 57.2 ± 7.3 kg, and 63.5% women, were randomized into eight groups, with each group comprising 12 participants. Blood samples were collected pre-and post-consumption to assess GI and GL values following established protocols.

**Results:**

The findings revealed that Phetchaburi’s Custard Cake exhibited the lowest GI and GL values (53.4 and 26.7, respectively), with progressively higher values observed in Saraburi’s Curry Puff (61.8 and 30.9), Nakhon Sawan’s Mochi (68.9 and 34.4), Suphan Buri’s Sponge Cake (75.9 and 38.0), Ayutthaya’s Cotton Candy (81.4 and 40.7), Prachuap Khiri Khan’s Pineapple Cheese Cake Biscuit (87.4 and 43.7), Chon Buri’s Bamboo Sticky Rice (109.3 and 54.7), and Lampang’s Crispy Rice Cracker (149.3 and 74.7), respectively.

**Conclusion:**

The study demonstrates that while Thai desserts exhibit a range of GI values, their GL values are uniformly high. It underscores the importance of disseminating GI and GL information to consumers, enabling them to make informed dietary choices and moderate their intake of these sugary delicacies.

## Introduction

The dietary glycemic index (GI), a concept introduced in 1981, is a critical measure indicating the impact of digested food on blood glucose levels (1). The glycemic load (GL) complements this by quantifying the change in blood glucose in relation to the carbohydrate amount consumed (2). Both indices are crucial for managing dietary impacts on blood sugar, especially for individuals needing sugar control or managing diabetes (3, 4).

Research on the GI and GL across the globe has been extensive. Studies have highlighted the role of GI in food choice, demonstrating how high-GI foods can rapidly increase blood glucose levels, impacting pancreatic function and escalating the risk of diseases like NCDs, metabolic syndrome, and cardiovascular disease (5–11).

In the United Kingdom, research on commercially available products revealed that most ready-to-eat meals had low-GI values, except for those with high mashed potato content (12). The Middle East, particularly the UAE, has seen studies reporting GI and GL values of traditional foods, providing dietary guidance for the local population (13). In Asia, investigations include the impact of processing on the GI of wheat flour in India (14) and the GI and GL values of traditional Chinese foods (15, 16).

In Thailand, research has primarily focused on rice and fruits. Studies have reported low GI in varieties of rice, including brown and germinated brown rice (17), and explored the relationship between sugar content and GI in Thai fruits (18–20). However, the GI and GL values of Thai desserts, often high in carbohydrates and sugar, remain understudied (21).

Thai desserts are renowned for their taste, vibrant colors, and cultural significance, representing the diverse heritage of Thailand’s provinces (22, 23). These desserts, popular among tourists, highlight regional culinary differences and include unique local ingredients (24). Their high simple carbohydrate sugar content may significantly impact gut microflora (25).

This study aimed to fill this gap by quantifying the GI and GL of popular Thai desserts, thus contributing to the international database. This information will help consumers make informed dietary choices, potentially reducing disease risk and aiding in weight management and insulin regulation. Additionally, the study will provide dietary recommendations based on the glycemic response of the desserts, ensuring safety through chemical and microbiological analysis before trials (21).

## Participants and methods

### Food identification

The selection of desserts for this study was based on a comprehensive survey conducted by the Tourism Authority of Thailand (TAT) across 76 Thai provinces (24). This survey identified a range of popular foods and desserts, from which 44 foods and 10 desserts were selected for further study based on their popular ratings.

For each of the 10 desserts, representing 10 distinct provinces from four major regions of Thailand, three brands (labeled as a, b, and c) were randomly selected. This selection process ensured a diverse representation of regional culinary traditions. The provinces and their respective desserts included the following:

- Northern region: Lampang’s Crispy Rice Cracker/Khao-Tan (P04), Kamphaengphet’s Grass Jelly/Chao-Guay (P03), and Nakhon Sawan’s Mochi/Mo-Ji (P05).
- Central region: Suphan Buri’s Sponge Cake/Sa-Lee (P10), Ayutthaya’s Cotton Candy/Roti-Sai-Mai (P01), and Saraburi’s Curry Puff/Ka-Ree-Puff (P09).
- Western region: Phetchaburi’s Custard Cake/Khanom-Mo-Kaeng (P07), and Prachuap Khiri Khan’s Pineapple Cheese Cake Biscuit/Pai-Sap-Pa-Ros (P08).
- Eastern region: Chonburi’s Bamboo Sticky Rice/Khao-Lam (P02).
- Southern region: Phatthalung’s Caramel/Ka-La-Mae (P06).

These desserts were sourced from a variety of shops across Thailand and chosen through a random selection process. The selection criteria for these shops were rigorously defined to ensure quality and authenticity. The criteria included the following:

1. Local production: Shops must exclusively produce and distribute dessert products within their province, ensuring the authenticity and regional specificity of the desserts.
2. Food Safety Certification: Shops must hold at least one food safety certificate from a recognized official agency. This could include a food quality assurance certificate from the Food and Drug Administration (FDA) within the Ministry of Public Health (Division of Consumer Protection) or a food safety certificate from the Public Health Pharmacy Group Provincial Public Health Office.
3. Product Labeling: The name and manufacturer of each dessert product must be clearly stamped on the product label, providing traceability and transparency.

From each selected shop, three different products of the same dessert type were obtained, amounting to a total of 30 brands. This approach aimed to capture the variety within each dessert category while maintaining a controlled and systematic sampling.

### Food contamination and nutrition value analysis

This stage of the study focused on a comprehensive analysis of ten products, each representing one from the three brands of the top 10 renowned Thai desserts, to evaluate food safety and nutritional value.

### Preservative analysis

To detect benzoic acid and sorbic acid, the selected dessert samples were subjected to the high-performance liquid chromatography method (HPLC). The samples were prepared by cutting them into small pieces, drying, and powdering; 10 g of each powdered sample was dissolved in a methanol and 0.01 M ammonium acetate water buffer (2:3 v/v), sonicated for 10 min for complete extraction, and centrifuged at 3,000 rpm for 5 min. The resulting supernatant was filtered through a 0.45-μm cellulose membrane syringe filter before being analyzed using on an Agilent 1,100 HPLC column. Concentrated samples were diluted further with the mobile phase for precision.

### Microbial contamination analysis

The microbial quality assessment involved checking for total aerobic plate count (APC), yeast, and mold (26). The methods used adhered to the FDA guidelines and were carried out by the Food Quality Assurance Service Center (FQA), a division of the Institute of Food Research and Product Development (IFRPD). Detected microorganisms in the dessert products were then identified at the FQA laboratory, which is accredited in food testing. This analysis ensures comprehensive safety evaluation in terms of microbiological content.

### Nutritional value analysis

Using an HPLC, the nutritional content of the desserts was analyzed to understand their health implications. This included quantifying key nutritional components to offer insights into the nutritional profile of these traditional Thai desserts.

### Ethical considerations

The research protocol for this study was meticulously reviewed and approved by the Ethics Committee for Human Research, Faculty of Medicine, Chulalongkorn University, Bangkok, Thailand (COA No. 1414/2021, IRB No.696/63). Prior to randomization, all subjects were thoroughly informed about the purpose, procedures, potential risks, and benefits of the study. To ensure informed decision-making, each participant provided written and signed informed consent, reflecting their voluntary agreement to participate after understanding all aspects of the study. This process was in strict compliance with the ethical guidelines stipulated in the Declaration of Helsinki of the World Medical Association and the International Conference on Harmonization Guidelines for Good Clinical Practice. Furthermore, to maintain transparency and accountability, the study protocol was registered with the Thai Clinical Trials Registry (TCTR20201008003) before enrolling the first participant.

### Study design, subjects, and blood sample collection

This investigation was structured as an open-label, randomized clinical trial designed to adhere to robust scientific standards and ensure the reliability of the results. The trial followed a systematic approach for participant eligibility determination, encompassing a comprehensive history taking, health check-up, and physical examination, assessment of waist circumference and body mass index (BMI), and blood laboratory tests.

**Figure 1:**
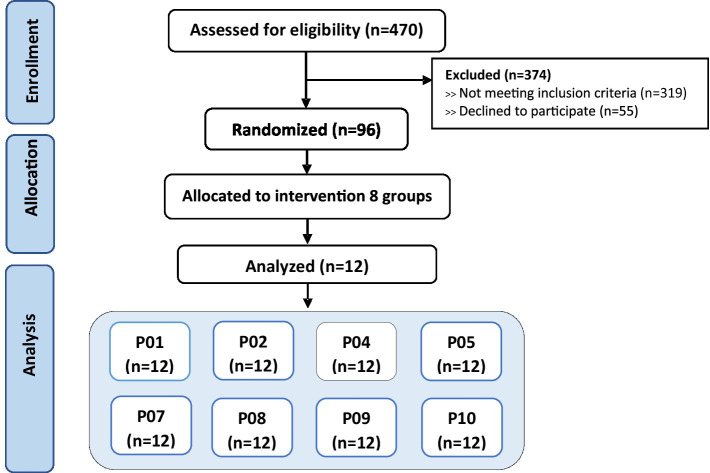
CONSORT flow diagram.

A total of 96 healthy Thai volunteers were enrolled in the study. Participants were Thai men and women aged between 18 and 45 years, in good general health with no chronic medical conditions. They had no history of metabolic disorders, such as diabetes mellitus, impaired glucose tolerance, metabolic syndrome, cardiovascular disease, kidney disease, or gastrointestinal diseases. Additionally, participants were not taking any regular medications that could affect glucose metabolism. Laboratory assessments confirmed that all participants had normal values for fasting blood sugar, total cholesterol, low-density lipoprotein (LDL), high-density lipoprotein (HDL), triglycerides, and HbA1c. Physical examinations showed normal findings, including appropriate waist circumference and BMI within the normal range according to Asian standards (18.5–22.9 kg/m^2^). Participants were excluded if they had any underlying medical conditions known to influence glucose tolerance or metabolism, a first-degree family history of diabetes mellitus, or abnormal laboratory values outside the normal reference ranges. Those who were taking medications impacting glucose levels, engaged in heavy smoking (more than 10 cigarettes per day), or excessive alcohol consumption were also excluded. Pregnant or breastfeeding women were not eligible to participate (Figure 1).

Participants were randomly assigned to one of eight dessert groups, with each group comprising 12 subjects (*n* = 12). Each participant received only one type of dessert to test. This random assignment ensured that each dessert was evaluated by 12 different participants. There were no dropouts during the study duration; all participants completed the trial as per the protocol.

Baseline characteristics of all subjects included normal fasting blood glucose levels. The protocol of the study drew inspiration from the methodologies described by Brouns et al. (27, 28) and incorporated methods suggested by the FAO/WHO for glycemic response studies (21). According to the FAO/WHO guidelines, for reliable GI determination, a test should be conducted on six or more subjects, while testing on ten subjects is recommended for greater statistical power and precision (27). Venous blood samples from each subject were collected and processed. Within 30 min of collection, the samples were centrifuged to separate plasma, which was then promptly delivered for blood glucose level analysis.

### Determination of glycemic index and glycemic load

**Figure 2:**
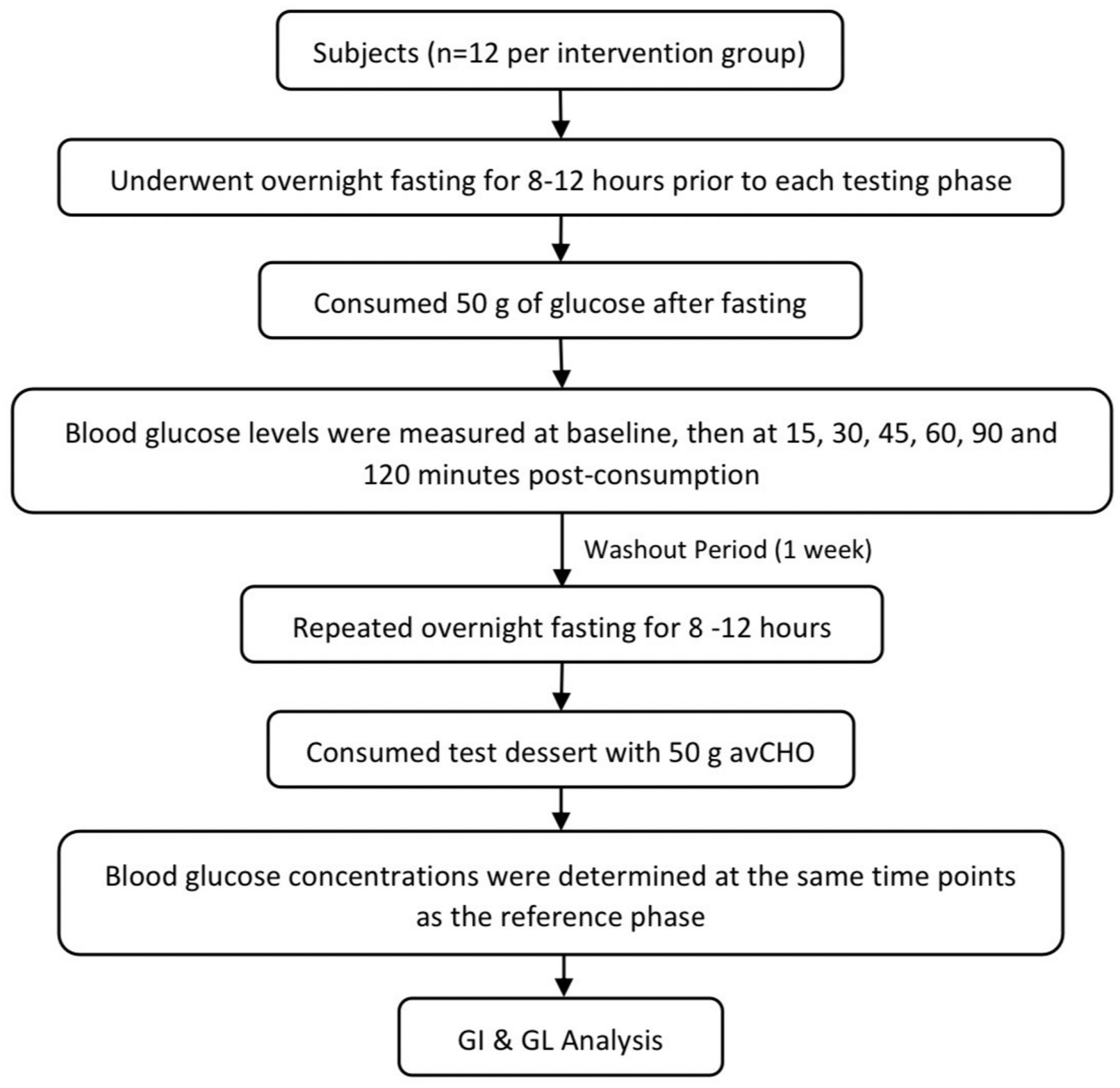
Schematic diagram of the glycemic index and glycemic load testing protocol.

Participants fasted overnight for 8–12 h before each test. Initially, a reference glucose solution (50 g) was administered, and venous blood samples were collected at intervals of 15, 30, 45, 60, 90, and 120 min post-consumption. Following a 1-week washout period, this procedure was repeated with the test desserts, each providing 50 g of available carbohydrates (avCHO). Blood samples were then drawn by registered nurses using sterile techniques. A volume of 1.5 mL of blood was collected at each time point and immediately sent for glucose analysis. Participants were divided into eight groups, each assigned a different dessert. Blood glucose levels post-dessert consumption were compared with the baseline glucose solution. The glycemic index was calculated by plotting the blood glucose response (area under the curve) against the baseline. This streamlined approach provided a direct comparison of the glycemic response to different Thai desserts, enabling the accurate calculation of their glycemic index and load (Figure 2).

### Calculations and statistical analysis

Descriptive statistics were employed to calculate the uptake of preservatives in desserts. The focus was on ensuring that preservative levels remained within the safe limit of <1,000 mg/kg as per regulatory guidelines. Microbial analysis involved total colony count data, expressed in colony-forming units (CFU) per gram of food. This analysis adhered to the quality criteria set by the Department of Medical Sciences, Ministry of Health (29). Results were presented as percentages relative to established standards (Table 1).

**Table 1:** Microbiological standards for selected Thai desserts by region.

Parameters	Colonies	Desserts	Product Code
Total number of bacteria/g Number of yeasts/g Number of molds/g	< 1 × 10^6^ < 1 × 10^3^ < 1 × 10^3^	Ayutthaya’s Cotton Candy Chonburi’s Bamboo Sticky Rice Phatthalung’s Caramel Phetchaburi’s Custard Cake	P01 P02 P06 P07
Total number of bacteria/g Number of yeasts/g Number of molds/g	< 1 × 10^5^ < 5 × 10^2^ < 5 × 10^2^	Lampang’s Crispy Rice Cracker Prachuap Khiri Khan’s Pineapple Cheese Cake Biscuit	P04 P08
Total number of bacteria/g Number of yeasts/g Number of molds/g	< 1 × 10^4^ < 1 × 10^2^ < 1 × 10^2^	Kamphaengphet’s Grass Jelly Nakhon Sawan’s Mochi Saraburi’s Curry Puff Suphan Buri’s Sponge Cake	P03 P05 P09 P10

Department of Medical Sciences, Ministry of Public Health, 2017.

The glycemic index (GI) was calculated using the incremental area under the curve (IAUC) method (30). This method involves plotting the blood glucose response over time and applying the trapezoid rule to calculate the area. The GI of each dessert was defined as the IAUC for that dessert, expressed as a percentage of the IAUC for 50 g of glucose, with all areas below baseline excluded. The final GI value for each dessert was derived from the average of results obtained from 12 participants and reported as mean ± SE. The formula used was as follows: GI = (IAUC dessert/IAUC reference glucose) × 100 (27).

The glycemic load (GL) of each dessert was calculated by considering both the GI value and the carbohydrate content per serving. Specifically, the GL was determined by multiplying the GI of the dessert by the amount of available carbohydrates in a 50 g serving (avCHO). The formula used for this calculation is as follows: GL = (GI of the dessert x avCHO in a serving (g))/100 (31).

For statistical analysis, the data were processed using IBM SPSS Statistics version 28. Prior to analysis, the normality of quantitative variables was assessed using the Kolmogorov–Smirnov test, which confirmed that the data were normally distributed (*p* > 0.05). Results were expressed as percentages, frequencies, and means with standard errors (mean ± SE). Statistical significance was established at a 95% confidence level (*p* < 0.05). To test the mean differences in baseline data of the subjects, a one-way ANOVA statistical test was applied (32).

## Results

### Chemical contamination

Among the 30 brands analyzed for chemical contamination due to preservative use, two brands, constituting 6.7% of the total, were found exceeding the permissible levels of preservatives (Table 2). Specifically, the levels of benzoic acid in brand P06c and sorbic acid in brand P10c surpassed the regulatory limit of 1,000 mg/kg, indicating a significant deviation from accepted food safety standards.

**Table 2:** Detected levels of preservatives in the dessert brands.

Product code	Brand	Benzoic acid (mg/kg)	Sorbic acid (mg/kg)
P01	P01a	22.94	ND
	P01b	112.04	ND
	P01c	481.59	ND
P02	P02a	768.95	ND
	P02b	ND	ND
	P02c	ND	ND
P03	P03a	ND	ND
	P03b	ND	ND
	P03c	ND	ND
P04	P04a	ND	ND
	P04b	ND	ND
	P04c	ND	ND
P05	P05a	ND	ND
	P05b	ND	ND
	P05c	ND	ND
P06	P06a	ND	ND
	P06b	ND	ND
	P06c	1,348.51*	ND
P07	P07a	22.44	ND
	P07b	82.61	ND
	P07c	ND	ND
P08	P08a	ND	ND
	P08b	ND	ND
	P08c	ND	ND
P09	P09a	ND	464.73
	P09b	ND	619.74
	P09c	ND	ND
P10	P10a	ND	ND
	P10b	ND	ND
	P10c	ND	4,448.00*

ND: Not detected. Values exceeding the prescribed standards are marked with an asterisk (*).

### Microbial contamination

Microbial contamination was detected in 12 of the 30 brands tested, accounting for 40% of the total. Specific concerns were identified with certain products. Six brands (P03a, P03b, P03c, P07a, P09b, and P10c) showed bacterial presence beyond acceptable levels. Yeast contamination was observed in four brands (P06a, P06b, P06c, and P03b). Two brands (P06a and P06b) were found to contain mold. Particularly, products P03 and P06 across all brands were deemed unsafe due to the presence of both bacteria and yeast, indicating significant hygiene issues. In addition to microbial concerns, excessive levels of preservatives were found in two brands (P06c and P10c), with benzoic acid and sorbic acid surpassing the safety threshold of 1,000 mg/kg. These findings highlight critical lapses in food safety and hygiene practices for certain brands of Thai desserts (Table 3).

**Table 3:** Microbial counts in Thai dessert brands.

Product code	Brand	Aerobic plate count (CFU/g)	Yeasts (CFU/g)	Molds (CFU/g)
P01	P01a	1.2 × 10^5^	1.6 × 10^2^	ND
	P01b	3.3 × 10^3^	ND	ND
	P01c	5.1 × 10^3^	ND	ND
P02	P02a	2.7 × 10^2^	ND	ND
	P02b	6.0 × 10	ND	ND
	P02c	ND	ND	ND
P03	P03a	1.8 × 10^8^	0.4 × 10	0.4 × 10
	P03b	7.4 × 10^7^	3.2 × 10^3^	ND
	P03c	1.9× 10^7^	1.1 × 10	0.8 × 10
P04	P04a	ND	ND	ND
	P04b	5.0 × 10^3^	ND	ND
	P04c	1.4 × 10^3^	2.8 × 10^2^	4.3 × 10^2^
P05	P05a	5.0 × 10	ND	ND
	P05b	5.0 × 10	ND	ND
	P05c	6.7 × 10^2^	ND	ND
P06	P06a	1.4 × 10^3^	3.1 × 10^3^	1.9 × 10^3^
	P06b	4.0 × 10^4^	2.9 × 10^3^	1.1 × 10^4^
	P06c	1.0 × 10^6^	1.9 × 10^3^	7.3 × 10^2^
P07	P07a	3.1 × 10^7^	ND	ND
	P07b	2.2 × 10^2^	ND	ND
	P07c	2.0 × 10	ND	ND
P08	P08a	ND	ND	ND
	P08b	4.5 × 10	ND	ND
	P08c	1.6 × 10^2^	ND	ND
P09	P09a	ND	ND	ND
	P09b	2.4 × 10^4^	ND	ND
	P09c	3.0 × 10	ND	ND
P10	P10a	9.5 × 10	ND	ND
	P10b	1.0 ×10^3^	ND	ND
	P10c	6.0 × 10^4^	ND	ND

ND: Not detected.

The evaluation identified eight Thai desserts that satisfied the set quality and food safety standards: Ayutthaya’s Cotton Candy (P01), Chon Buri’s Bamboo Sticky Rice (P02), Lampang’s Crispy Rice Cracker (P04), Nakhon Sawan’s Mochi (P05), Phetchaburi’s Custard Cake (P07), Prachuap Khiri Khan’s Pineapple Cheese Cake Biscuit (P08), Saraburi’s Curry Puff (P09), and Suphan Buri’s Sponge Cake (P10). The nutritional values of these selected desserts were meticulously analyzed to calculate their GI and GL, essential for assessing the amount consumed by the subjects (Table 4). This nutritional analysis covered total and available carbohydrates, serving sizes, sugar, fat, protein content, and total energy for each dessert. The gathered data facilitated the precise calculation of the GI and GL, which is crucial for evaluating the effects of these desserts on blood glucose levels. Such evaluations are particularly relevant for dietary recommendations aimed at individuals managing their blood sugar levels.

**Table 4:** Serving size and nutritional value of Thai desserts.

Brand	Thai dessert	Total CHO g/100 g	Available CHO (g)	Serving Size (g)	Sugar (g)	Fat (g)	Protein (g)	Total energy (Kcal)
P01a	Ayutthaya’s Cotton Candy	56.1	50.0	89.2	15.8	8.1	4.9	292.5
P02c	Chon Buri’s Bamboo Sticky Rice	39.6	50.0	126.2	17.0	9.9	4.7	308.4
P04a	Lampang’s Crispy Rice Cracker	73.4	50.0	68.2	15.4	10.9	2.6	308.4
P05a	Nakhon Sawan’s Mochi	62.5	50.0	79.9	18.6	10.4	5.2	314.3
P07c	Phetchaburi’s Custard Cake	26.0	50.0	192.2	40.2	14.8	10.5	375.4
P08a	Pineapple Cheese Cake Biscuit	74.5	50.0	67.1	24.4	6.9	3.0	274.1
P09c	Saraburi’s Curry Puff	51.1	50.0	97.9	15.5	15.1	8.2	368.8
P10a	Suphan Buri’s Sponge Cake	42.6	50.0	117.5	32.5	5.0	7.5	275.0

CHO = Carbohydrates.

### GI and GL of desserts

The study involved 96 healthy subjects divided into eight groups, with a balanced distribution of male and female participants. The subjects had an average age of 31.8 ± 5.7 years, with women comprising 63.5% of the group, and an average weight of 57.2 ± 7.3 kg. The mean Body Mass Index (BMI) across participants was 20.9 kg/m^2^. Analysis of biochemical baseline data indicated no significant variance between groups (ANOVA; *p* > 0.05), suggesting homogeneity in baseline characteristics among the groups (Table 5).

**Table 5:** Baseline characteristics of subjects by intervention group.

Characteristic	Total (*n* = 96)	P01 (*n* = 12)	P02 (*n* = 12)	P04 (*n* = 12)	P05 (*n* = 12)	P07 (*n* = 12)	P08 (*n* = 12)	P09 (*n* = 12)	P10 (*n* = 12)
Women *n* (%)	61 (63.5%)	6 (50.0%)	9 (75.0%)	9 (75.0%)	7 (58.3%)	7 (58.3%)	9 (75.0%)	7 (58.3%)	7 (58.3%)
Age (years)	31.8 ± 5.7	33.6 ± 4.7	31.7 ± 7.2	33.4 ± 6.7	31.0 ± 6.2	30.3 ± 5.0	31.3 ± 5.9	32.4 ± 5.7	30.9 ± 5.6
Weight (kg)	57.2 ± 7.3	56.9 ± 8.1	55.1 ± 7.1	56.2 ± 6.5	58.1 ± 7.6	57.8 ± 8.3	55.2 ± 7.6	59.2 ± 7.4	59.3 ± 6.6
Height (cm)	164.9 ± 7.8	164.3 ± 8.7	163.2 ± 8.4	164.1 ± 7.2	165.3 ± 8.6	165.9 ± 7.4	162.2 ± 9.1	167.0 ± 7.1	167.1 ± 6.1
BMI (kg/m^2^)	20.9 ± 1.3	20.9 ± 1.4	20.6 ± 1.3	20.8 ± 1.2	21.2 ± 1.2	20.9 ± 1.6	20.9 ± 1.1	21.2 ± 1.8	21.2 ± 1.4
Waist (cm)	74.7 ± 5.0	75.8 ± 5.3	73.2 ± 5.2	73.4 ± 4.5	74.7 ± 4.9	75.9 ± 4.9	72.3 ± 5.3	75.9 ± 4.5	76.6 ± 5.0
Bangkok residence n (%)	64 (66.7%)	9 (75.0%)	6 (50.0%)	6 (50.0%)	10 (83.3%)	9 (75.0%)	7 (58.3%)	8 (66.7%)	9 (75.0%)
Current smokers *n* (%)	0.0	0.0	0.0	0.0	0.0	0.0	0.0	0.0	0.0
Current alcohol *n* (%)	30 (31.3%)	3 (25.0%)	3 (25.0%)	3 (25.0%)	5 (41.7%)	4 (33.3%)	3 (25.0%)	4 (33.3%)	5 (41.7%)
Physical activity *n* (%)									
No activity	31 (32.3%)	3 (25.0%)	4 (33.3%)	4 (33.3%)	4 (33.3%)	4 (33.3%)	3 (25.0%)	4 (33.3%)	5 (41.7%)
Light	55 (57.3%)	8 (66.7%)	7 (58.3%)	8 (66.7%)	5 (41.7%)	6 (50.0%)	9 (75.0%)	7 (58.3%)	5 (41.7%)
Moderate	10 (10.4%)	1 (8.3%)	1 (8.3%)	0.0	3 (25.0%)	2 (16.7%)	0.0	1 (8.3%)	2 (16.7%)
FBS (mg/dl)	84.6 ± 4.9	88.2 ± 4.9	84.7 ± 3.3	84.8 ± 5.8	83.7 ± 5.2	82.6 ± 4.6	84.8 ± 3.1	83.9 ± 4.9	83.9 ± 6.1
Hb1Ac (%)	5.0 ± 0.3	5.2 ± 0.3	5.1 ± 0.3	5.0 ± 0.3	4.9 ± 0.3	4.9 ± 0.4	5.0 ± 0.3	4.9 ± 0.3	4.9 ± 0.4
Total cholesterol (mg/dl)	171.0 ± 20.3	179.8 ± 17.2	179.2 ± 17.9	168.3 ± 24.1	168.3 ± 18.9	164.8 ± 21.3	178.8 ± 18.1	164.3 ± 20.4	164.8 ± 20.4
Triglyceride (mg/dl)	60.3 ± 23.0	69.1 ± 27.5	68.5 ± 28.3	57.3 ± 25.6	53.9 ± 14.5	59.9 ± 23.4	61.3 ± 22.6	60.8 ± 22.9	51.2 ± 16.2
LDL (mg/dl)	100.8 ± 27.4	115.8 ± 21.4	108.9 ± 22.8	94.3 ± 29.9	93.3 ± 31.3	94.8 ± 27.6	107.4 ± 18.4	94.8 ± 32.7	97.3 ± 29.6
HDL (mg/dl)	61.4 ± 14.1	58.3 ± 14.1	63.3 ± 12.0	67.4 ± 17.3	62.0 ± 14.1	58.7 ± 9.9	67.0 ± 18.6	56.9 ± 11.6	57.9 ± 12.8
ALT (U/L)	15.8 ± 5.4	17.3 ± 9.4	14.9 ± 4.9	13.8 ± 4.5	16.0 ± 2.8	18.33 ± 7.2	14.3 ± 4.5	16.4 ± 3.6	15.4 ± 3.1
AST (U/L)	17.8 ± 3.2	16.8 ± 4.3	18.0 ± 3.6	17.4 ± 3.2	18.5 ± 1.8	18.2 ± 3.4	17.3 ± 3.7	18.7 ± 3.2	17.8 ± 2.7
BUN (mg/dl)	10.8 ± 2.9	11.1 ± 2.7	10.5 ± 3.0	11.0 ± 3.2	11.1 ± 3.0	10.3 ± 2.7	10.6 ± 3.2	10.8 ± 3.1	11.2 ± 2.9
Creatinine (mg/dl)	0.8 ± 0.2	0.8 ± 0.2	0.7 ± 0.2	0.7 ± 0.2	0.8 ± 0.2	0.8 ± 0.2	0.7 ± 0.2	0.8 ± 0.2	0.8 ± 0.2

Mean ± SD.

**Figure 3:**
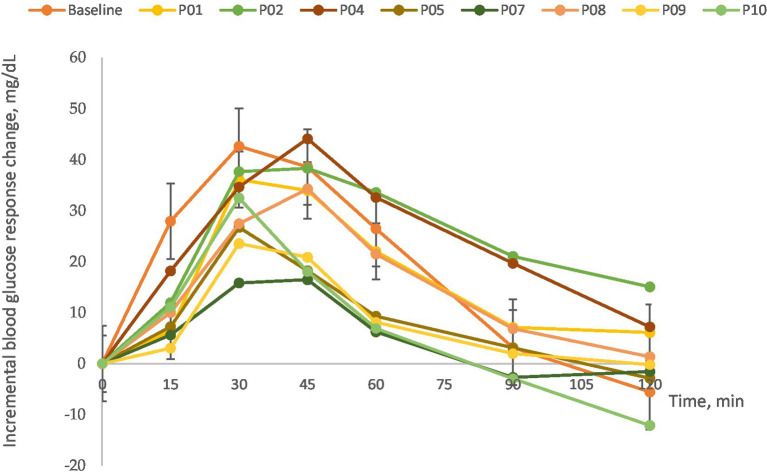
Dynamic blood glucose response to Thai desserts over time.

Figure 3 illustrates the mean changes in blood glucose concentrations from fasting baseline levels across the eight test desserts. It was observed that blood glucose levels in subjects peaked between 30 and 45 min following consumption of the selected Thai desserts and normalized by 120 min. This pattern underscores the glycemic response to the desserts, providing insights into their GI and GL values.

The GI and GL values determined for the selected Thai desserts are presented in Table 6. The calculated GI values for the eight desserts varied significantly, ranging from a low of 53.4 to a high of 149.3. According to the classification by Brand-Miller et al. (34), GI values are categorized as low (≤ 55), medium (56–69), and high (≥ 70). Based on this system, Phetchaburi’s Custard Cake had a low GI (53.4), Saraburi’s Curry Puff, and Nakhon Sawan’s Mochi were categorized as medium (61.8 and 68.9, respectively), while the remaining desserts, including Suphan Buri’s Sponge Cake (75.9), Ayutthaya’s Cotton Candy (81.4), Prachuap Khiri Khan’s Pineapple Cheese Cake Biscuit (87.4), Chon Buri’s Bamboo Sticky Rice (109.3), and Lampang’s Crispy Rice Cracker (149.3), were classified high. When examining the GL values based on a 50-g available carbohydrate serving, all desserts were classified as high according to the GL classification system by Venn et al. (33) with GL values exceeding 20.

**Table 6:** Sorted glycemic index and load of Thai desserts based on experimental and standard serving sizes.

Product code	Thai dessert	Experimental portion (g)	GI	GL (experimental portion)	Standard serving size (g)	CHO (g per standard serving)	GL (per standard serving)
P07	Phetchaburi’s Custard Cake	192.16	53.4 ± 14.4	26.7	140	36.4	19.5
P09	Saraburi’s Curry Puff	97.94	61.8 ± 14.7	30.9	30	15.3	9.5
P05	Nakhon Sawan’s Mochi	79.99	68.9 ± 12.3	34.4	40	25.0	17.2
P10	Suphan Buri’s Sponge Cake	117.50	75.9 ± 15.9	38.0	30	12.8	9.7
P01	Ayutthaya’s Cotton Candy	89.19	81.4 ± 10.5	40.7	40	22.4	18.3
P08	Prachuap Khiri Khan’s Pineapple Cheese Cake Biscuit	67.11	87.4 ± 16.8	43.7	40	29.8	26.0
P02	Chon Buri’s Bamboo Sticky Rice	126.20	109.3 ± 19.9	54.7	80	31.7	34.6
P04	Lampang’s Crispy Rice Cracker	68.15	149.3 ± 24.8	74.7	40	29.3	43.8

Mean ± SE.

## Discussion

The determination of reliable GI and GL values for iconic Thai desserts such as Phetchaburi’s Custard Cake, Saraburi’s Curry Puff, and others is vital not only for researchers but also for the general populace. In our study, the groups of subjects tested exhibited no significant statistical differences in baseline characteristics, ensuring the validity of the GI measurements. Our results indicated that most of the Thai desserts evaluated had medium-to high-GI values. Suphan Buri’s Sponge Cake had a GI of 75.9, which is comparable to that of white bread (GI > 70). In fact, five of the desserts studied—Suphan Buri’s Sponge Cake, Ayutthaya’s Cotton Candy, Prachuap Khiri Khan’s Pineapple Cheese Cake Biscuit, Chon Buri’s Bamboo Sticky Rice, and Lampang’s Crispy Rice Cracker—were classified as high-GI food (GI > 70). Desserts with medium GI values (GI 55–69) included Saraburi’s Curry Puff and Nakhon Sawan’s Mochi. The only low-GI dessert (GI < 55) identified in our study was Phetchaburi’s Custard Cake.

The observed GI differences are likely due to the diverse ingredients and their ratios, particularly the types and amounts of carbohydrates and sugars used in the desserts (35). Despite its higher sugar content, Phetchaburi’s Custard Cake exhibited a lower GI, suggesting lower glucose absorption possibly due to the presence of fats and proteins that delay digestion. Conversely, Lampang’s Crispy Rice Cracker, despite a lower sugar content, showed a higher GI. This can be attributed to its composition—primarily sticky rice, topped with cane sugar and fried, which may alter the starch structure, making the glucose more rapidly absorbable. These findings underline the importance of considering the whole food matrix, including types and amounts of carbohydrates, cooking methods, and additional ingredients, in understanding the GI values of foods (36).

Further analysis of the GL values for standard servings reveals a crucial aspect of dietary impact. The standard servings of Thai desserts, significantly smaller than the experimental portions, still present high GL values, underscoring the importance of portion control in dietary planning. Given the elevated GL values observed even in smaller, more realistic servings, this information is essential for consumers, particularly those managing glycemic responses. Moderation in the consumption of these desserts, guided by an understanding of both GI and GL values, is advisable to mitigate potential health risks associated with elevated blood glucose levels.

From a public health perspective, the high-GI and GL values observed in several Thai desserts highlight a significant concern. Regular consumption of high-GI foods can lead to rapid spikes in blood glucose levels, increasing the risk of developing type 2 diabetes, obesity, and cardiovascular diseases. Therefore, implementing strategies that inform and educate consumers about the glycemic impacts of the foods they consume is imperative.

Introducing GI and GL information on food labels can empower consumers to make informed dietary choices. By understanding the glycemic effects of desserts, individuals can moderate their intake of high-GI foods and opt for alternatives with lower glycemic responses. Educational campaigns are essential to raise awareness about the importance of GI and GL in managing blood glucose levels and overall health. Such initiatives can guide consumers in interpreting GI and GL values and incorporating this knowledge into their dietary habits.

Policymakers could consider regulations that mandate the disclosure of GI and GL information on packaged foods, especially for products with high carbohydrate content. Setting guidelines for maximum allowable sugar content in desserts could contribute to reducing the glycemic load of population. Encouraging food manufacturers to reformulate products to achieve lower GI values by modifying ingredients or preparation methods can also be beneficial.

Collaborating with food producers and culinary experts to modify traditional dessert recipes by reducing sugar content or incorporating ingredients with a lower glycemic impact can help decrease the GI and GL of these foods without compromising cultural heritage. For example, incorporating whole grains or fiber-rich ingredients may lower the glycemic response.

Regarding food safety, microbial contamination was found in 12 of the 30 dessert brands, with bacteria, yeast, or mold detected in products across various brands. Alarmingly, all brands of certain desserts such as grass jelly and caramel were found to be contaminated, indicating a systemic issue that transcends brand variations. Additionally, two brands, including caramel and sponge cake, were found to have chemical contamination due to excessive levels of preservatives, exceeding the threshold of 1,000 mg/kg. The prevalence of contamination highlights the critical need for comprehensive safety practices. As demonstrated in prior studies, sources of contamination can include production processes, packaging, and environmental factors (37). Compliance with established microbiological quality guidelines such as the FAO/WHO Codex (CAC/GL21-1997) is essential to safeguard consumer health (38).

A GI database for particular Thai desserts will be instrumental in guiding dietary choices, particularly for individuals with diabetes or those managing their glycemic response. Desserts high in carbohydrates, such as Ayutthaya’s Cotton Candy and Prachuap Khiri Khan’s Pineapple Cheese Cake Biscuit, significantly influence blood glucose levels, which can strain pancreatic function due to the rapid insulin production needed to process the influx of sugar. Understanding the GL in the context of portion sizes is crucial; our findings suggest that consuming these desserts in moderation is advisable to prevent adverse health outcomes.

Our findings underscore the need for a multifaceted approach to address the public health challenges posed by high-GI desserts. Mandating the inclusion of GI and GL information on packaging can help consumers make healthier choices by providing clear nutritional information. Public health campaigns should educate the population about the impacts of high-GI foods and how to manage glycemic responses through diet, enhancing consumer awareness and encouraging healthier eating habits. Governments can implement regulations to limit sugar content in desserts and promote the availability of lower GI options, thereby supporting public health initiatives. Additionally, collaborating with food manufacturers to reformulate products can reduce the glycemic impact without sacrificing cultural significance, fostering industry cooperation for healthier food options.

By combining these strategies, it is possible to reduce the risks associated with excessive consumption of high-glycemic foods and promote healthier dietary patterns among the Thai population. Integrating GI and GL information into dietary guidelines and nutrition education programs will empower individuals to make informed choices, ultimately contributing to better health outcomes.

A comparison of glycemic responses (GI and GL) between male and female participants was not conducted in this study. Each dessert group consisted of 12 participants with unequal numbers of men and women, with male participants ranging from 4 to 6 per group. The small and uneven sample sizes limited the statistical power to detect potential sex differences in glycemic responses. Consequently, our results reflect the combined responses of all participants regardless of sex. Future studies with larger and more balanced samples are recommended to explore whether sex differences influence the glycemic impact of these desserts.

## Conclusion

The Thai desserts analyzed in this study, including Phetchaburi’s Custard Cake, Saraburi’s Curry Puff, Nakhon Sawan’s Mochi, Suphan Buri’s Sponge Cake, Ayutthaya’s Cotton Candy, Prachuap Khiri Khan’s Pineapple Cheese Cake Biscuit, Chon Buri’s Bamboo Sticky Rice, and Lampang’s Crispy Rice Cracker, were found to have a range of GI values from low to high, and uniformly high GL values. Given these findings, it is advisable for consumers to be mindful of the portions they consume, particularly in terms of proteins, fats, and carbohydrates, to prevent adverse health effects related to diabetes, obesity, or cardiovascular diseases. For individuals aiming to maintain health by managing blood glucose levels, selecting desserts with lower GI may be beneficial. The data on GI and GL values from this study can serve as a valuable resource for consumers, researchers, and dietitians making informed choices about Thai desserts. Furthermore, this information could influence positive changes in dessert consumption habits, enhancing public health and product quality. Additionally, these insights may guide the refinement and development of local Thai desserts, bolstering their role in promoting Thailand’s culinary tourism.

## Data Availability

The raw data supporting the conclusions of this article will be made available by the authors, without undue reservation.
